# The All-Stakeholders-Considered Case for Corporate Beneficence

**DOI:** 10.1007/s10551-022-05224-9

**Published:** 2022-10-27

**Authors:** Gastón de los Reyes

**Affiliations:** Glasgow Caledonian New York College, 64 Wooster St., New York, NY 10012 USA

**Keywords:** Beneficence, Corporate responsibility, Pandemics, Social contract theory

## Abstract

In ways accentuated by the global coronavirus pandemic, corporations constitute vital instruments of the acts of beneficence needed by the people of the world to make progress in public health and increase collective and individual well-being. This article contributes to understanding the variety of moral forces that may lead corporations to commit acts of beneficence, including Friedman’s business case for corporate beneficence, the duty of beneficence as developed by business ethicists, and Dunfee’s social contract account of corporate obligation. Whereas Mejia recently contributed to scholarship on corporate beneficence by expressly adopting shareholder primacy’s conception of corporate governance, this article embraces a stakeholder-oriented, managerialist picture of corporate governance. I extend the literature on beneficence by incorporating what I argue is the intuition underlying Dunfee’s contractualist formula of minimal contribution, namely that management’s duty to do good is awakened and unshackled to the extent management judges the corporation can afford to commit acts of beneficence, all stakeholders considered. The all-stakeholders-considered case for corporate beneficence compels management to act, I argue, when inaction would undermine the moral integrity of managers personally committed to promoting the well-being of humanity.

In ways accentuated by the global coronavirus pandemic, corporations constitute vital instruments of the acts of beneficence needed by the people of the world to make progress in public health and increase collective and individual well-being. The global experience during the present context confirms that “corporate power... must be brought to bear on certain social problems if they are to be solved at all” (Andrews, 1972)—no corporate action, for example, no mRNA COVID-19 vaccines.^1^ I am motivated in this article to address a specific question about corporate beneficence raised by the current pandemic: the fact that Moderna and the Pfizer–BionTech partnership that own lifesaving mRNA vaccine technology are refusing to share the knowledge so more lives can be saved *as soon as possible*, not later when the first facility to reverse engineer the Moderna drug finds out whether Moderna has been sincere in professing to renounce its right to enjoin the manufacture of a copycat drug (Roelf, 2021). Thus, I want to ask much the same question Dunfee (2006) posed in retrospect about the obligation of those pharmaceutical companies controlling components of the triple-vaccine therapy that started saving lives in the 1990s. After millions had died and continued to die in sub-Saharan Africa of HIV but before those companies began sharing the recipe and otherwise helping to save millions more lives, Dunfee wanted to know: “do firms with unique competencies for rescuing victims of human catastrophes have special obligations?”.

Experience confirms what Adam Smith (1776) theorized: acts of “corporate beneficence” (Mejia, 2021b, p. 426) need not be motivated by a moral commitment towards beneficence but can be “aimed at increasing the financial returns of shareholders or the company’s reputation” (p. 426). The pursuit of a corporation's interests has been normatively grounded as a feature of management’s moral responsibility by Milton Friedman’s (1970, p. 123) powerful endorsement of the business case for corporate beneficence. My interest in this article is to look at the various arguments that have been given for moral forces that *should* drive acts of corporate beneficence so as to perspicaciously show how my contribution is different and conceptually advantageous as against the best known categories of arguments in business ethics: (i) the moral commitment to shareholders conceived by Friedman to answer to the business case for corporate beneficence and (ii) the moral duty of beneficence asserted by Kantian theorists to sometimes motivate management to act outside a clear business case (see Table 1). I identify with the latter because, as I will show below, I find the Kantian point of view in business ethics illuminates a distinctive moral force that is very much worth building upon and extending.

**Table 1:** Survey of four moral forces that drive acts of corporate beneficence

	1. Friedman’s business case for corporate beneficence	2. The duty of beneficence (extant business ethics literature)	3. The duty to normative shareholders to commit acts of beneficence	4. The statement of minimal moral obligation	5. The all-stakeholders-considered case for corporate beneficence
Representative proponent	Friedman (1970)	See section “Friedman's Business Case for Corporate Beneficence”	Mejia (2021a, 2021b)	Dunfee (2006)	This article
Spectrum from shareholder primacy to stakeholder-oriented	Shareholder primacy	Corporation and/or management faces stakeholder-oriented duty of beneficence whose sway is limited by business imperatives	Shareholder primacy (incorporating “normative shareholders’” duties to stakeholders)	Corporation/management faces SMMO that is multi-stakeholder-oriented with special deference to shareholders	Stakeholder-oriented with managerialist (company-centered) conception of corporate governance (Bower & Paine, 2017)
Theorist’s normative intention/logic	Setting forth duties that apply to management irrespective of personal commitments of management or shareholders	Setting forth duties that apply to management/corporations irrespective of personal commitments of management or shareholders	Setting forth duties that apply to management irrespective of personal commitments of management or shareholders	Setting forth duties that apply to corporations by virtue of various pertinent contingencies, including corporation’s mission and prior social initiatives	Clarifying the conditions that should grip beneficent management to commit acts of corporate beneficence
The moral ground	Agency duty to shareholders who (are assumed to “generally”) desire maximal wealth	The duty of beneficence of the corporation and/or management	Agency duty to what shareholders should want companies to do to discharge their duty of beneficence *qua shareholders*	The multi-stakeholder social contract of business corporations and management’s role responsibility in furtherance thereof	A multi-stakeholder-oriented managerial commitment to beneficence
Imperative force	Pursue the end of maximal shareholder value through corporate beneficence as a means	Seek to promote the well-being of others	“[I]f shareholders need to coordinate their efforts to discharge a duty emerging from the corporate activities, the manager should fulfill the duty on their behalf” (2021a)	Steer the corporation to fulfill its social contract as detailed by the SMMO	Promote the well-being of others by committing acts of corporate beneficence responsive to the all-stakeholders-considered case

My conceptual strategy for making a novel contribution to this literature is threefold. First, I adopt Mejia’s (2021b) theoretical strategy and expressly embrace a specific and concrete conception of corporate governance because it brings resolution and definition to my normative inquiry into management’s corporate responsibility to commit acts of corporate beneficence. Mejia (2021a, 2021b) makes a novel contribution to the substantial business ethics literature on the duty of beneficence by starting from Friedman’s principal-agent model and management’s corresponding moral commitment to above all act loyally towards shareholders’ interests. Based on this point of view, Mejia argues that the operative moral duty of beneficence that ought to compel management actually inheres in *shareholders*—not managers or the corporation as such—so that “if shareholders need to coordinate their efforts to discharge a duty emerging from the corporate activities, the manager should fulfill the duty on their behalf” (2021a, pp. 11–12).

Second, to figure out how to conceptualize the moral forces in question, I develop an original interpretation of Dunfee’s (2006) idiosyncratic view of the moral obligation to commit acts of beneficence on behalf of the corporation. Dunfee views the corporation as the moral agent in question, subject to the imperative force of the social contract he argues corporations ought to discharge. Though I will argue that the plausibility of Dunfee’s argumentation is undermined by its statute-like detail and logic, I seek to demonstrate that the reasoning with which Dunfee answers his question—“do firms with unique competencies for rescuing victims of human catastrophes have special obligations?”—elucidates *how* the interaction of multi-stakeholder considerations can present itself to management with compelling enough force to yield acts of corporate beneficence.

The third key theoretical move that animates my account is making an argument that is normative in a materially different sense than prevails in business ethics generally and in the duty of beneficence literature in particular. I do not frame my argument and the resulting case for corporate beneficence around what management ought to do *in abstraction from what they really do care about*. Rather, I am trying to discern the moral forces available to managers who *already do* positively embrace a commitment to promote the well-being of others—taking for granted a managerialist, stakeholder-oriented conception of corporate governance. These two theoretical moves, together with Dunfee’s lucid intuitions about the bare minimum that pharmaceutical companies ought to do in global pandemics, position me to give much more definition than has been given in the beneficence literature as to *when* the force of beneficence compels action. Consequently, to contrast with the “business case” for corporate beneficence, I call the distinct argumentative moral force I develop in my extension of the beneficence literature the “all-stakeholders-considered case” *for corporate beneficence*.

The manuscript proceeds as follows: In the next section, I motivate my choice to deploy a managerialist, stakeholder-oriented conception of corporate governance that is more expansive about corporate purpose than Friedman’s “shareholder primacy” (Hsieh, 2017). In the section that follows “Friedman’s Business Case for Corporate Beneficence,” I recapitulate Friedman’s account of the moral force that ought to respond to the business case for corporate beneficence, since business ethicists in beneficence and Dunfee (2006) characterize their views in relation to Friedman’s version of shareholder primacy. The next section, “Beneficence in Business Ethics,” reviews the business ethics literature on a moral force whose legitimacy Friedman expressly rejects—management’s or the corporation’s duty of beneficence. I do so to position my account in relation to the theoretical advantages and also the limitations in this scholarship that warrant further theorizing. Then, in “Dunfee’s Statement of Minimal Moral Obligation,” I develop Dunfee’s account to highlight the intuitions that make his reasoning compelling. In “The All-Stakeholders-Considered Case for Corporate Beneficence,” I propose an innovative way to integrate the duty of beneficence’s motivation from within a manager’s moral integrity with the argumentative multilateralism of Dunfee’s view (Table 2) to yield the all-stakeholders-considered case for corporate beneficence. The next section addresses the limitations of my account. I close in the conclusion by arguing that the boundary condition in my view vis-à-vis the beneficence literature—that management positively embrace a commitment to promote the well-being of others—in fact constitutes the distinctive and novel strength of the account.

**Table 2:** Structure of Dunfee’s argument

Section	Title	Reasonable criticisms Dunfee wishes to answer
II.	Firms with Unique Human Catastrophe Rescue Competencies	That the duty interrupts moral free space too frequently/substantially
III.	A Statement of Minimal Moral Obligation (SMMO)	*All the criticisms*
IV.	Implementing the SMMO	That implementing the duty is infeasible
V.	Justification for recognizing special obligations on the part of firms with unique human catastrophe rescue competencies	That there is no good reason to make an exception to moral free space for rescue
VI.	Does the SMMO impose an unfair or inappropriate burden on alternative donees?	That the SMMO will hurt other stakeholders with morally significant interests
VII.	Does the SMMO impose an unfair or inappropriate burden on global pharmaceuticals?	That the SMMO is unwarranted and too burdensome
VIII.	Is the SMMO too modest?	That the SMMO is too modest
	Or, instead, is it too radical?	That the SMMO is too radical

## The Call for Stakeholderist Managerialism

Over a century the pendulum of social legitimacy has swung back and forth between two views. The first is managerialism, which takes for granted as unproblematic “managerial control of the corporation” (Hendry, 2001). Second is the normative conception of corporate governance that makes management loyal first and foremost to shareholders’ financial interests based on a property rights justification, as iconically formulated by Friedman (1962, 1970). This second view, often labeled shareholder primacy (Hsieh, 2017), has been enormously influential in the business ethics literature—frequently as foil (Heath et al., 2010, pp. 443–446)—and also for managerial practice (Bower, 2008, p. 273; Bower & Paine, 2017). It has been institutionalized with corporate governance reforms that increasingly led executives to be compensated with stock incentives, starting in the 1970s (Dobbin & Jung, 2010).

Notwithstanding its overwhelming influence upon business schools and business itself (Bower & Paine, 2017; Khurana, 2010), shareholder primacy has been challenged over many decades (Andrews, 1972; Bower & Paine, 2017; Dodd, 1932; Khurana, 2010; Kovvali & Strine, 2022; Shaw, 1916; Stout, 2012). Long before Friedman, 

in what is sometimes called the Great Debate . . . Adolph Berle (1931) and Merrick Dodd (1932) squared off in the pages of the *Harvard Law Review*, with Berle arguing for shareholder primacy and Dodd supporting a broader purpose that includes secure employment, quality products for customers, and contributions to the good of society” (Harrison et al., 2019, p. 1226).

Whereas shareholder primacy holds that the shareholder comes first (Berle, 1931; Friedman, 1970), “managerialism” (Enteman, 1993; see Learned et al., 1965) understands corporate governance in a constitutionally distinct way. This view is “company-centered” (Bower & Paine, 2017) and stakeholder-oriented with “a broader purpose that includes secure employment, quality products for customers, and contributions to the good of society” (Harrison et al., 2019). Through this point of view, therefore, I am seeking to learn about the moral forces that may move management to commit acts of beneficence by expressly adopting a managerialist, stakeholder-oriented picture of corporate governance.

The managerialist picture behind the seminal strategy casebook, *Business Policy: Text and Cases* (Learned et al., 1965), paints the relation between management and shareholders totally differently from Friedman’s shareholder primacy:

The notion that the shareholder of a large, publicly held corporation is its owner grows increasingly indefensible. He owns shares, which represent so small a commitment on his part that he may through the mechanisms of the stock market shed it instantly. Management, to whom has come a virtually permanent delegation of authority for continuing direction of the publicly held corporation, is still bound to run the company to serve shareholder interest. But neither by law nor by custom does it have the simple obligation to pursue maximum profit (Andrews, 1972, p. 137).

The 1980s brought Freeman’s (1984) articulation of a stakeholder theory of corporate governance and strategy that has given the business ethics classroom the evergreen “Friedman versus Freeman” debate (see Agle et al., 2008, pp. 162–166; Freeman, 2010, p. 7). Should one embrace the view that the purpose of management is to maximize the profits of shareholders or to manage the corporation for stakeholders broadly? The influence of Freeman’s conception of stakeholder management in business schools and business cannot be overstated, and stakeholder theory has in this century become a legitimate approach for scholarship in strategic management (e.g., Harrison et al., 2010; Tantalo & Priem, 2016).

In recent years, legal and business scholars have refreshed Andrews’s managerialist attack upon the premises that reinforce shareholder primacy’s picture of corporate governance (Bower & Paine, 2017; Hart, 2010; Stout, 2012). Bower and Paine argue that shareholder primacy is “confused as a matter of corporate law and a poor guide for managerial behavior,” reasoning that “public company shareholders have few incentives to consider, and are not generally viewed as responsible for, the effects of the actions they favor on the corporation, other parties, or society more broadly” (Bower & Paine, 2017, p. 52). Stout’s (2012) book-length debunking of “the shareholder value myth” questions legal, economic, and empirical arguments for shareholder primacy to conclude that “many of the problems we see in the corporate sector today are the unintended consequences not of corporations as such, but of a mistaken *idea* about corporations: the idea that they ought to be run to maximize shareholder value as measured by share price” (pp. 104–105).

The legitimacy of stakeholder-oriented managerialism as a picture of corporate governance was catapulted forward by the 2019 issuance of the Business Roundtable’s (BRT) “Statement on the Purpose of the Corporation” (Business Council of Institutional Investors, 2019; Business Roundtable, 2019; Edelman, 2019; Firestone, 2019; Govindarajan & Srivastava, 2020; Harrison et al., 2019; Ramaswamy, 2020; Sorkin, 2019). Signed by 181 CEOS, the BRT’s Statement distances its members from the BRT’s past principles of corporate governance, which transmitted shareholder primacy’s stipulation that corporations “exist principally to serve their shareholders” (Business Roundtable, 2019). In place of a loyalty that revolves around shareholders, the signatories state that they “endeavor every day to create value for all our stakeholders” (Business Roundtable, 2019), in the spirit of the managerialism advocated by Andrews and Freeman.^2^ As verbalized by Airbnb, for example, the sense of mission is broadly stated to “benefit all our stakeholders over the long term” (Ramaswamy, 2020).

The point of this brief review was not to settle the longstanding debate but rather (i) to motivate the importance of assessing the moral forces that may compel corporate beneficence from this standpoint and (ii) to highlight where a stakeholder-oriented and managerialist conception of corporate governance diverges from the premises of shareholder primacy.

## Friedman’s Business Case for Corporate Beneficence

To understand the relevance and importance of the duty of beneficence literature (section “Beneficence in Business Ethics”) as well as Dunfee’s (2006) idiosyncratic proposal (section “Dunfee’s Statement of Minimal Moral Obligation”) and my own constructive approach (section “The All-Stakeholders-Considered Case for Corporate Beneficence”), the starting point is Friedman’s (1970) articulation of shareholder primacy in terms of management’s obligation to always *act* “in the interests of shareholders” (p. 33). In Friedman’s view, this compels management to promote what he assumes shareholders generally desire: “as much money as possible while conforming to the basic rules of the society, both those embodied in law and those embodied in ethical custom” (p. 33). This leads Friedman to impugn managers who commit corporate resources to social purposes (p. 123). Nevertheless, notice that since for Friedman the ultimate measure of management’s moral duty is shareholders’ financial interest, beneficence is morally permitted, nay, required, when committing acts of corporate beneficence promotes shareholders’ long-term interest in as much money as possible.

As Friedman explains, “it may well be in the long-run interest of a corporation that is a major employer in a small community to devote resources to providing amenities to that community or to improving its government” (Friedman, 1970, p. 123). Why? Well, it “may make it easier to attract desirable employees, it may reduce the wage bill or lessen losses from pilferage and sabotage or have other worthwhile effects” (p. 123). Given its touchstone of moral duty, the force behind the Friedman doctrine’s *business case for corporate beneficence* is equally disposed to motivate the sales activity that management conventionally entertains to make shareholders wealthy (Amis et al., 2020, p. 500).^3^ This includes developing vaccines for COVID-19 and HIV and manufacturing personal protective equipment for sale in the market in response to the signals of the “price mechanism” (Hayek, 1945). It also includes taking as much money as possible from government [e.g., Moderna has received $6 billion in payments from the United States government (Saltzman, 2021)] and finding how to avoid taxes (Denning, 2019).

The critical handicap of the market channel of the business case for corporate beneficence is that markets are mute when it comes to individuals too impoverished to register (sufficient) economic demand—no matter how urgent, dire, and easily remedied their needs (e.g., Chance & Deshpandé, 2009). Any demand signal transmitted to the market has to be presented by surrogate buyers—be they governments, non-governmental organizations (NGOs), or corporations themselves—advocating in the market on behalf of those in need. In responding to pandemics, such institutions do as a matter of fact often step in to purchase and deploy goods and service on behalf of others (e.g., Thomas, 2020), and normative theories in other domains, such as political theory (White, 2000), may bring light to what society should expect of these institutions given the role responsibilities of those in charge (compare Roscoe, 2020). Governments are positioned not only to act as buyers in the market but in all countries they also directly control important capabilities to commit acts of beneficence, such as the capacity to distribute payments that cut childhood poverty in the United States by over half in 1 year (DeParle, 2021).

The practical importance of inquiring into alternative moral forces that may produce acts of corporate beneficence owes to the reality that such market surrogates frequently fail to appear—at least in the numbers required to save lives and prevent hardship. This has not stopped corporations in either of the article’s focal cases—the HIV crisis and the current COVID-19 pandemic—from bringing their core competences (Prahalad & Hamel, 1990) to bear to some meaningful extent. The overarching inquiry in this article is into the variety of alternative moral forces that may help explain corporate responsiveness to commit acts of beneficence, other than Friedman’s business case for corporate beneficence, to situate the place and distinction of the all-stakeholders-considered case.

## Beneficence in Business Ethics

Business ethicists have theorized about the duty of beneficence as a moral force that can drive corporate beneficence outside the business case. Overwhelmingly, they have adopted a Kantian conception of morality to do so, where duty emanates from reason, and morality concerns imperatives that categorically bind rational, human agents (Korsgaard, 1999, pp. 9–10). Like Mejia (2021b), I will frame my contribution as a constructive yet critical extension of this literature. My first objective in this section is to review the key characteristics of the scholarship for building blocks in my account. Second, I will show that this literature’s theorizing as developed is too vague to yield a managerially prescriptive imperative (that clearly compels *acts* of corporate beneficence). In contrast with Mejia (2021b), this literature assesses beneficence as a feature of corporate and managerial responsibility that does not hinge on inferences about the “normative shareholder’s” duties. In this regard, my account more directly extends the literature in beneficence by focusing on managerial beneficence, whereas Mejia translates shareholders’ duty of beneficence into a moral command that, like shareholder primacy, binds managers without regard to their personal proclivities.

I begin in the sub-section “The Obligation to Act with Beneficence” by briefly setting forth the Kantian roots of the duty of beneficence and the significance of its status as an “imperfect” duty. This will position me to show why Hill (1971) concludes that the duty of beneficence, while imperfect, sometimes makes it “non-discretionary” (Mejia, 2021a, 2021b) to act with beneficence.^4^ It is against the backdrop of Hill’s account, consistent with Mejia (2021b, p. 434), that I review business ethicists’ assessments of the duty of beneficence in the sub-section “Business Ethicists on the Duty of Beneficence.”

### The Obligation to Act with Beneficence

In the Kantian tradition, the “duty of beneficence” (Bowie, 2017; Hsieh, 2017) is construed to be “imperfect” rather than “perfect” because the categorical imperative of one’s moral commitment “allow[s] a latitude for choice not permitted by perfect duties” (Hill, 1971, p. 56; see Mejia, 2021b, pp. 428–429). To wit, in the case of beneficence’s duty to promote the well-being of humanity, moral agents reasonably and necessarily have to balance and integrate the decision to act (or not) from beneficence alongside other commitments, including a healthy concern for one’s own well-being and self-interests (Hill, 1971, p. 62; Robinson, 2019, p. 48; Smith, 2012, p. 71). This conceptual structure means the duty’s generalizable guidance to act sums up to the slippery, “Sometimes promote the happiness of others” (Hill, 1971, p. 71).

When it comes to an imperfect duty like beneficence, “the moral law ‘can prescribe only the maxim of actions, not actions themselves’” (Hill, 1971, p. 57, quoting Kant, 1964, p. 49). For beneficence, the maxim involves the commitment to act to promote the well-being of others. Acting in moral concert with the duty of beneficence, consequently, involves judgments of *congruence* and *harmony* rather than straight-edged, dualistic rules of consistency (cf. Aristotle, 1999, p. 145, 1137b30; compare Christensen’s (2010, pp. 50–51) “marginal cost mistake”). The congruence required by the duty of beneficence resides within one’s will, meaning that, however, one chooses to act fits sincerely alongside a practiced commitment to promote the well-being of humanity. That is why Kant (1964) says that “no determinate limits can be assigned to what should be done, the duty has in it a play-room for doing more or less” (p. 12; see Hill, 1971, p. 56; cf. Andrews, 1972, p. 143). Humane and caring people can uphold their integrity of will and honor beneficence *without* necessarily writing checks to every charity that could use their help—the moral agent also faces a duty to figure out how to take care of themselves*.* In sum, “the principle of beneficence is to be construed as allowing me considerable freedom to pursue my own happiness provided that I adopt and act on a maxim to promote the happiness of others also” (Hill, 1971, p. 60).^5^

Notwithstanding the foregoing—and this represents a crucial insight for the position I will articulate and defend in section “The All-Stakeholders-Considered Case for Corporate Beneficence”—Hill finds that the form of Kant’s account may nevertheless render acts of beneficence “obligatory because it is the only way, given the agent’s past record, to satisfy the limited demands of the principle” (p. 71). What can render an act of beneficence obligatory is the *incongruence* between (x) choosing *inaction* while (y) *also* embracing the maxim of beneficence in practice. In the jargon I will use to convey the motivational quality of this incongruence, the agent *can’t afford not to* act with beneficence while also plausibly reaffirming their commitment to live morally, which this person agrees means promoting the well-being of humanity.

The key variables that can render beneficence non-discretionary in Hill’s insightful analysis (and the “can’t afford not to” framing I wish to use) are (i) the integrity of the agent whose moral standing demands congruence between their acts and their moral maxims and (ii) the factual context and history that figure into the “agent’s past record.”^6^ An important conceptual contribution of my analysis is extending Hill’s approach to the domain of corporate responsibility, where people—management—assume role responsibility to manage the corporation (Goldman, 1980). My strategy for extending Hill is to expressly recognize that what matters in the moral balance is not so much the manager’s past but rather (iii) the past record of the corporation whose acts management enacted (Schrempf-Stirling et al., 2016) and enacts as part of (iv) the agent’s role responsibilities *qua* manager (see section “The All-Stakeholders-Considered Case for Corporate Beneficence”).

### Business Ethicists on the Duty of Beneficence

In section “Business Ethicists on the Duty of Beneficence,” my objective is to demonstrate that the majority view in business ethics (prior to Mejia) holds that the duty of beneficence is (x) a binding duty, but (y) one with significant limits that render it (z) always discretionary. The literature is, therefore, vague about the imperative’s prescription to act through the corporation, providing limited insight about the conditions that convert this moral force into realized acts of corporate beneficence. I am able to rely on recent reviews of the literature by Bowie (2017), Hsieh (2017), Dubbink (2018), and Robinson (2019) to summarize pertinent features of the literature.

The first point is to recognize that business ethicists assert that the duty of beneficence is always binding and not optional. Smith (2012) wrote to develop Bowie’s (1991, 1999) seminal treatments of Kantian business ethics, and according to Bowie’s (2017) more considered view, Smith gets it right: “the duty of beneficence is always a duty” (p. 166). Dubbink (2018) reiterates Kantian presuppositions in holding that “[t]aking the principle of beneficence into account may never be overlooked” (p. 7). Robinson’s (2019) recent book on imperfect duties in business provides extensive discussion of Kantian theory with a persuasive derivation of the maxim of beneficence as a categorical imperative: “We cannot reasonably make the universal claim that everyone can remain independent of the beneficence of others. We must therefore adopt” beneficence as a maxim (p. 30). The duty of beneficence “demands a serious and continuous commitment to promoting the good of others” (Mejia, 2021b, p. 428).

The second key point I wish to highlight is the way business ethicists have attended to the practicalities of managing a business corporation in expounding upon the duty of beneficence. Robinson’s (2019) derivation of beneficence as a required component of morality dictates that “we ought to help others pursue their own ends *where and when we can*,” “*within* practical limitations” (p. 30, emphasis added).^7^ In this vein, Bowie (2017) addresses the appropriate scope of corporate beneficence by conceptualizing the duty as fitting *within* a CSR program that is sized to support a profitable corporate strategy:

My way of putting it is that Kant’s duty of beneficence requires that CSR be included as an essential element of corporate strategy and that it be implemented in terms of that strategy. It is not CSR instead of profits, or no CSR if profits are adversely affected. Rather, it is a type of CSR that is incorporated into a profitable long-term strategy for a publicly held firm (p. 167).^8^

Smith’s (2012) formulation similarly appeals to the potential congruence of profitable corporate strategy and beneficence: “[a] socially responsible corporation is one that does not shirk, but thoughtfully and creatively integrates a concern for the well-being of stakeholders with its overarching strategic endeavors” (p. 70). Smith says this framing is designed (like section “Dunfee’s Statement of Minimal Moral Obligation”) to “respond[] to the concern that a beneficent or socially responsible corporation cannot do what corporations are designed to do well, that is, to remain competitive and enhance profitability for investors” (p. 70). The resulting conception is motivational yet restrained: “beneficence provides us with an alternative that does not posit responsible corporations as those that enhance human welfare in the aggregate, but as those that develop operational plans that effectively integrate concern for others into their commercial relationships” (pp. 71–72). Seconding Smith’s concern that the corporation’s purpose should not inflate to beneficence at large (2012), Robinson (2019) cautions that doing so would be counterproductive: “If broad obligations of beneficence were applied to management, but without practical limits, then management could hardly function in rationing and utilizing resources so as to provide goods and services to the general public” (p. 46). Bowie (2017) goes so far as to hold that “the perfect duty to seek shareholder profit” overrides any duty to *act* on beneficence (p. 169, see Hill, 1971, p. 57).

The picture of beneficence as a duty that binds management and yet accommodates business imperatives like “remain[ing] competitive and enhanc[ing] profitability for investors” (Smith, 2012, p. 70) has understandably led business ethicists to conclude that beneficence is impotent to generate tractable prescriptions (compare Mejia, 2021b, p. 428). In Dubbink’s (2018) unequivocal formulation: “In a specific situation, it is simply never wrong—after consideration—not to make the principle of beneficence the determining principle of action” (p. 8). Posing the question whether “the content of the duty of beneficence can ever be fully specified,” Smith (2012) “think[s] we must answer in the negative” (p. 72).

Ohreen and Petry (2012) take inspiration from Hill’s (2002) more recent work and allude to Singer’s (1972) famous example of the moral call to rescue a child in a shallow pond. They speculate that “we might be obligated... where circumstances dictate immediate action, especially when there is little sacrifice on our part” (p. 374; see Mejia, 2021b, p. 434). Ohreen and Petry gesture towards a more general theory of beneficence’s imperative in the rescue context: “Strict obligations arise, perhaps, when circumstances dictate that it would be reasonable for others to expect me to help and where there is wide discrepancy between meeting the needs of others and costs incurred” (pp. 374–375).

Two suggestions from this last account help introduce Dunfee’s (2006) argument for the contractualist formula of obligation that I present next. First, Ohreen and Petry’s speculation about when and why beneficence might ever obligate specific acts suggests [as does Mejia (2021b, pp. 434–435)] that beneficence could become obligatory when it is required to rescue someone. Second, their formula (unlike Mejia’s) postulates that the duty’s grip also depends on (reasonable) stakeholder expectations as well as the “costs incurred” in giving effect to rescue. Note that both of these ideas resonate and fit conceptually as elaborations of the majority view in the business ethics literature I reviewed, and they will be central to the extension I develop in section “The All-Stakeholders-Considered Case for Corporate Beneficence” based on insights from Dunfee (2006).

## Dunfee’s Statement of Minimal Moral Obligation

Friedman’s (1970) business case for corporate beneficence rejects any social responsibility of the corporation, beyond living up to the moral duty to prioritize shareholders’ financial wealth interest. Whereas business ethicists have theorized about beneficence without bringing into the foreground the distinction between the moral responsibilities of the corporation and those that belong to management, Dunfee (2006) occupies a unique space in the discourse about the moral forces that drive acts of corporate beneficence. He does not square off against Friedman’s delegitimization of managers’ personal commitments to beneficence (as the literature on beneficence could be read to do), but rather rejects Friedman’s contention that “[a] corporation is an artificial person and in this sense may have artificial responsibilities, but ‘business’ as a whole cannot be said to have responsibilities” (1970, p. 33). For Dunfee, corporations are social institutions that ineluctably find themselves morally bound by “extant social contracts” (Dunfee, 1991). Whereas Dunfee and his coauthor Donaldson are best known for their fairly technical Integrative Social Contracts Theory (ISCT) (Donaldson & Dunfee, 1999), the sense of social contract limned by Dunfee (2006) is not technical and hearkens back to the basic intuition behind Dunfee’s pre-Donaldsonian account: “Extant social contracts, deriving from communities of individuals, constitute a significant source of ethical norms in business” (1991, p. 23).

I wish to excavate Dunfee’s approach for insights with which to extend the beneficence literature because his argument incorporates facts about the real world, namely, the contingent institutions that he thinks influence extant social contracts about corporate beneficence. These empirical contingencies bring his account a level of specificity that the duty of beneficence literature as a whole lacks. In particular, his Statement of Minimal Moral Obligation (SMMO) takes notice of the prevalence and positive legitimacy of social investment budgets among pharmaceutical companies, and these institutions provide Dunfee an ingenious way to fulfill his project to define a quasi-enforceable obligation of the corporation: “If a firm has chosen to engage in social initiatives, then one can argue that it has voluntarily accepted a social role, at least within the boundaries of its prior actions” (p. 204).

### The Empirical Conditions and Conceptual Framing of Dunfee’s (2006) Project

In 1996, scientists announced that a “cocktail” combining three different antiretroviral drugs (triple-drug therapy) could treat HIV successfully (Vella et al., 2012). Merck, followed by Glaxo-Wellcome (today, GlaxoSmithKline) and other companies, owned one or more components of the lifesaving cocktail, and they each also controlled vast capabilities for the manufacture and distribution of drugs worldwide.^9^ In the ensuing decade, sub-Saharan Africa was overwhelmed by an HIV crisis of disastrous and enduring proportions (Haas et al., 2018). Dunfee encapsulates the jaw-dropping scale of the human catastrophe:

In 2003, 1.2 million people died of AIDS in sub-Saharan Africa while another 3 million became newly infected. In 2001, a total of 28.5 million people were infected with HIV/AIDS in the region. In 2004, 2.4 million sub-Saharan Africans died of the disease while no more than one million received treatment with antiretroviral drugs. In the seven sub-Saharan countries where the prevalence of AIDS is greater than 20 percent, the average life expectancy is 13 years lower than it would be in the absence of AIDS (p. 187).

Not surprisingly, pharmaceutical companies with lifesaving patents found themselves, as Dunfee (2006) writes in the opening sentence of his article, “under extreme pressure to increase substantially their efforts to mitigate the AIDS catastrophe in sub-Saharan Africa” (p. 185). Certainly, people and governments in these countries were generally not wealthy (or insured) enough to afford the $10,000 per year being charged in the United States for triple-drug therapy (Chance & Deshpandé, 2009).

For Dunfee as a business ethicist, the conceptual quandary of the case of the HIV pandemic arises from Donaldson and Dunfee’s (1999) premise, reiterated by Dunfee (2006), that “firms have moral free space to decide the scope and nature of their commitment to social issues” (p. 190). Dunfee’s stated goal is to carve out a narrow exception with the SMMO that “imposes a highly circumscribed restraint” on the “moral free space” of corporations (p. 190) to pursue purely economic objectives, and he does so by developing an account that “promises to retain the core of shareholder primacy” (Hsieh, 2009, p. 554). In claiming that “managers are morally permitted and at times, required, to devote corporate resources to alleviate human misery even if this comes at the expense of shareholder interests” (Hsieh, 2009, p. 554), Dunfee follows Donaldson’s (1992) general view that “whatever duties corporations may have to aid the deprived... are duties whose performance is not required as a condition of honoring the concept of corporate responsibility,” while allowing that “[e]xtraordinary conditions are capable of creating exceptions to the principle” (p. 281, n. 13).

### The Statement of Minimal Moral Obligation

The potential for Dunfee’s article to provide new insight into the duty of beneficence comes from the way he interprets the minimal obligation, or duty, to get shaped by facts about the corporation’s institutional environment.^10^ Not only does Dunfee, the social contract theorist, assume and embed in his analysis of corporate responsibility the shareholder-oriented forms of corporate governance prevalent in global pharmaceutical companies (e.g., stock incentives for executives), he also takes notice of and (opposed to Friedman’s rejection of moral responsibilities for corporations) endorses the legitimacy of the fact that the pharmaceutical corporations of concern “already engage in voluntary social initiative programs” (p. 186).

With this picture in mind and his focus on the HIV crisis in sub-Saharan Africa, Dunfee’s first conceptual move is to head off shareholder primacy’s charge that the minimal obligation will be overdemanding for shareholders (Dubbink, 2018), given (as he assumes) that there will always be unmet human needs a company could help with. Dunfee wants to clarify that his argument will not create any duty for corporations to respond to “the ordinary types of social needs that corporations have historically considered as part of their charitable obligations” and that he is “focusing on devastating, overwhelming instances of human need” like those resulting from a pandemic (p. 187). Dunfee, therefore, infers a social contract that limits the trigger of the SMMO to companies with a “unique human catastrophe rescue competency” (p. 187), where a “catastrophe” implies that hundreds of thousands of people are being presently impacted with “severe injury, deprivation or death” (p. 188). What distinguishes corporations with a “unique competency” is that “*no other firm has a greater competency*” (p. 188, emphasis in the original).

The Statement’s text (p. 186, p. 190) consists of three legalistic sentences. Whereas the second two are carefully drafted to “satisfy most reasonable critics” (p. 194) and forestall the key objections Dunfee feels compelled to address in his article (see Table 2), the first sentence is open-ended and hortatory: “Firms possessing a unique human catastrophe rescue competency have a moral obligation to devote substantial resources toward best efforts to aid the victims of the catastrophe” (p. 190). Why, though, do corporations have this obligation? The SMMO itself does not give an obvious answer, and this represents one of the fundamental limitations with Dunfee’s approach (that the Kantian duty of beneficence redresses).^11^

The next two sentences of the SMMO establish the minimum dollar value of the corporation’s obligatory contribution with formulas that leave aside the human catastrophe in the world and instead look to the corporation’s existing commitments to shareholders and other stakeholders.

Unless financial exigency justifies a lower level of investment, they should devote, at a minimum, the largest sum of (1) their most recent year’s investment in social initiatives, (2) their 5-year average of investment in social initiatives, (3) their industry’s average investment in social initiatives or (4) the average investment in social initiatives by firms in their home nation (p. 190).

With respect to the pharmaceutical firms driving Dunfee’s inquiry, the applicable measure of capability deployment would be based on the company’s actual (1 above) or average trailing (2) social investment budget, and I will focus on the framing of the funding obligation under (1) and (2), so as to leave aside complications raised by alternatives (3) and (4).^12^ Notice that by assessing the bounds of the obligation in proportion to the corporate social investment budget, rather than the moral intensity (Jones, 1991) of the calling of urgent need,^13^ Dunfee mandates a social contribution level that “fall[s] within the range that has already been accepted by the firm” (p. 204), preparing him to answer the concern that the SMMO is too radical (Table 2).^14^

By further conditioning the size of the contribution required on the financial health of the company (“Unless financial exigency justifies a lower level of investment…”), the SMMO formulates a variable minimal obligation that is sensitive to a shareholder advocate’s worry (reasonable or not) that Dunfee’s proposal would threaten to undermine the corporation’s financial well-being. The example given to illustrate the rationale of this carve-out is Merck’s Vioxx liability, which according to Dunfee implies that it “may not be able to maintain the level of contributions it has made in the recent past” (p. 193). The message of the SMMO to shareholders, and to the managers who answer for and to them, is that financial exigencies can and do override the demands of the SMMO in whole or in part (like Bowie’s perfect duty to pursue shareholder profits).^15^

## The All-Stakeholders-Considered Case for Corporate Beneficence

Dunfee devotes his article’s theoretical efforts to fending off arguments that go against the grain of making the corporate contributions demanded by the SMMO (Table 2), and by doing so he generates deep insight into how multi-stakeholder management translates into discursive, imperative form. I now seek to operationalize his arguments to structure a cornerstone for the all-stakeholders-considered case for corporate beneficence set forth herein. I begin in the first sub-section, “What a Corporation Can Afford to Do, All Stakeholders Considered,” by showing that the moral intuition explaining Dunfee’s choices about the SMMO’s contingent requirements revolves around one common denominator: what management *can afford* to commit towards acts of beneficence when a global pandemic is decimating people’s lives.

My affirmative contribution to the beneficence literature comes in the next sub-section, “What Management Can’t Afford Not To Do For Moral Integrity,” through a conceptual merger of (i) Dunfee’s hub-and-spoke conception of what a corporation can afford to do with (ii) the Kantian logic of imperfect duty that roots acts of beneficence in the heart of well-integrated human agency. The value of the all-stakeholders-considered case is bringing into relief the conditions that awaken and reasonably set loose the will to act on behalf of the corporation in lifesaving ways—from the standpoint of management who adopt a managerialist, stakeholder-oriented conception of their role responsibility and already embrace a commitment to promote the well-being of others. In the final sub-section, “The mRNA Case for Corporate Beneficence,” I apply my normative account to the moral stakes as presented by the case that opened the article involving the corporations that own the mRNA vaccines, Moderna and Pfizer–BioNtech, before the ongoing global pandemic.

### What a Corporation Can Afford to Do, All Stakeholders Considered

Dunfee’s articulation of the minimal obligation to commit acts of corporate beneficence combines a variety of apparently diverse considerations—(1) the size of a corporation’s social investment budget, (2) the compelling rationales for existing beneficiaries of such social investment, and also (3) the financial exigencies the company might be facing at a point in time. Each of the three concerns—budget size, existing beneficiaries, and exigencies—factors into the way the SMMO’s formula variably regulates the minimum contribution that Dunfee asserts management should make, once the company is tapped by the social contract (because of its comparative advantage to respond to human catastrophe). That much is clear from the text of the SMMO. What my discourse analysis of Dunfee’s article showed (see Table 2) is that the implicit regulatory principle behind the variability of the SMMO is keeping the minimal obligation *within levels the corporation can afford as a matter of managing diverging stakeholder claims, with special deference to shareholders* (cf. Klein et al., 2019).

In taking a position on what these corporations *can afford* (my verbiage for the regulatory principle that explains the conditions he includes in the SMMO), Dunfee expressly relies on the positive legitimacy of social investment budgets that companies have already established (p. 204). Notice that management *can afford* corporate beneficence on this reading not because it does not cost anything or promises shareholder value at the margin (it most likely does not), but rather because the norms defining stakeholder claims create space within which management can afford the “latitude” (Andrews, 1972, p. 143) to redeploy corporate resources to pandemic response.

Rather than making the business case, Dunfee relies on the position occupied by the corporation in relation to shareholders but also to the reasonable norms that exist to attend to the well-being and interests of other stakeholders. Dunfee’s approach is not reducible to corporate rationality about reputation management or otherwise managing for sustainable competitive advantage. The moral labor in his account is being done by the asserted reasonability of the norm in question—the SMMO—given the institutions at hand and the corporation’s relation to them (see Dworkin, 2000; Cohen, 2009): it “does not seem radical to impose on a firm a duty whose basic costs fall within the range that has already been accepted by the firm” (Dunfee, 2006, p. 204).^16^ Financial profitability projections in a public corporations’ disclosures could be literally unaffected by the shifting of the social investment budget from one deployment to another,^17^ and this accounting fact gives gravitas to the kind of argument that Dunfee thinks management can defensibly present to “shareholders [who] should have known about the firm’s policies when they purchased their shares” (Dunfee, 2006, p. 192).

**Fig. 1:**
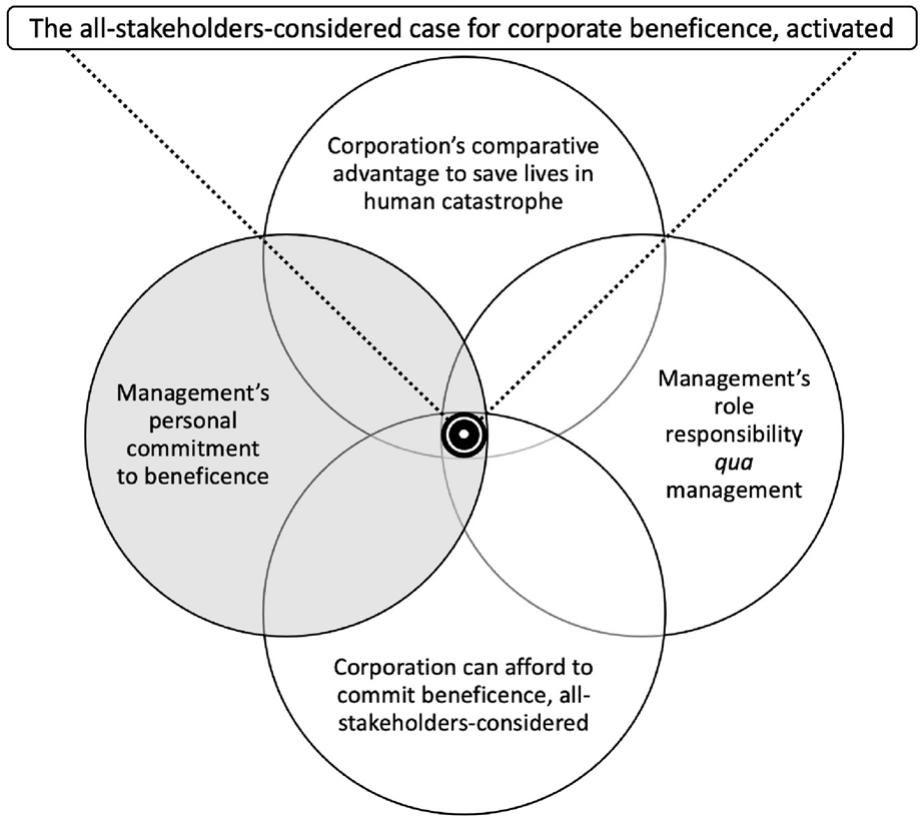
The All-Stakeholders-Considered Case for Corporate Beneficence, Activated

In trying to pin down minimal obligations as he does, perhaps Dunfee was too stingy in his assessment of what management can afford to do all stakeholders considered; perhaps conversely he was too flippant about shareholders’ financial interests. What I wish to draw from Dunfee is not his personal take as to what pharmaceutical management can or cannot afford to do in responding to a pandemic, but rather his underlying intuition about the existence of a decisional space within which (i) the corporation may enjoy a comparative advantage to save lives in a deadly global pandemic, (ii) the corporation could afford to commit acts of beneficence that will save lives from human catastrophe, and (iii) management enjoys enough latitude to so act on the corporation’s behalf inside the bounds of its role responsibility, *qua* management. Figure 1 demarcates this space.

What exactly *can* a corporation afford to do, all stakeholders considered? My objective in this article is not to answer this question but rather to argue that the imperative force yielded through this question comes to legitimately compel acts of corporate beneficence. The managerialist standpoint is critical in allowing “the voluntary restraint of short-term profit maximization” to be legitimately entertained because “the managers of a corporation [judge] that their powers are ultimately subject to public expectations that extend beyond the stockholders' interest in profit” (Andrews, 1972, p. 135).

Though I will not be offering an algorithm to cash out what a corporation can afford to do, all stakeholders considered, in this or that case, I would like to align myself with stakeholder theory’s mindset and application of a similarly qualitative standard to capture what the strategic quest in business is all about. For Freeman (2010), “the very heart of capitalism and its entrepreneurial spirit is in figuring out how to meet the demands of customers, suppliers, employees, communities, and financiers, so that all win” (p. 8). Finding how all stakeholders win as a check and channel for strategy is an aspirational way to confirm that the corporation can afford to go ahead and act in furtherance of stakeholder needs. Perhaps more importantly, Freeman stresses that the spirit of the stakeholder-oriented enterprise is to adapt to social transformation—as no doubt wrought by the COVID pandemic—remaining open and curious about new ways to comprehend stakeholder relationships:

when there is dissonance, the time is ripe to try and find a reframing of the basic business proposition so that more stakeholders win continuously over time. Stakeholders that are difficult to please, critics, employees who push back, even conflicts of values, all can be sources of value creation, when approached with the “no tradeoffs” mindset of managing for stakeholders (2010, p. 9).

Since the stakeholder-oriented processes undertaken by managerialist corporations are open-ended, adaptive, and dynamic, I cannot take a position in advance on the kind of engagement and calculations management needs to pursue before concluding the corporation can afford to act (compare Scholz et al., 2019, pp. 332–334). 18

 What is indispensable to the commitment to find what the corporation can afford to do is the strategic mindset that inspires stakeholder theory generally:

Serving all your stakeholders is the best way to produce long term results and create a growing, prosperous company. . . . Let me be very clear about this: there is no conflict between serving all your stakeholders and providing excellent returns for shareholders. In the long term it is impossible to have one without the other. However, serving all these stakeholder groups requires discipline, vision, and committed leadership (Freeman, 2010, p. 9, quoting George, 2003).

Excellence in corporate strategy is more than maximizing profits at the expense of the public—“[t]he very nature of capitalism itself is putting together a deal, or a contract, or a set of relationships among stakeholders so that all can win continuously over a long period of time” (p. 7). Management may thus find latitude, within the scope of its role responsibilities, to answer the special calling landing on companies with a comparative advantage to save lives—if the corporation can afford to help. The conceptual task now is to argue that management’s beneficence kicks in to activate the all-stakeholders-considered case for corporate beneficence.

### What Management Can’t Afford Not to Do for Moral Integrity

What converts the space where the corporation *can afford* to act (and doing so is consistent with management’s responsibility *qua* management) into the imperative to act is the force of management’s personal integrity. Think of moral integrity like a strong magnet that repels its opposite pole—which is acting (or embracing beliefs) inconsistently with the precepts of one’s integrity. When management is confronted by the strong call to save lives only its corporation could and concludes the corporation can indeed afford to act, the personal commitment to promote the well-being of others repels inaction. The all-stakeholders-considered case for corporate beneficence is activated (Fig. 1), consistent with a Kantian theory of moral integrity (see section “The Obligation to Act with Beneficence,” see also Korsgaard, 1997), because when the corporation *can afford* to act, management *can’t afford not to*, at least not without sacrificing their moral integrity.

The magnetic kind of force I reference is the commitment to personal integrity and beneficence that compels action when that is “the only way, given the agent’s [circumstances], to satisfy the limited demands of the principle, ‘Sometimes promote the happiness of others’” (Hill, 1971, p. 71). On my account, management comes to face a moral imperative when the corporation can afford beneficence, all stakeholders considered, because management find themselves able to answer, and by implication unable to deny, the call for capability deployment without putting into question the authenticity of (i) their personal commitment to beneficence or (ii) their sense of responsibility *qua* management (compare Dunfee, 2006, pp. 185–186). By virtue of their personal commitments and responsibilities, the external call of need that is unanswered by markets compels management to ask, “if not now, when?” And when management concludes as a matter of considered stakeholder engagement and analysis and in virtue of its role responsibility that the corporation can afford the acts of beneficence in question the imperative to so act vests. The prescriptive thrust of my argument says to management who on reflection admit to the personal commitment to advance the well-being of others: Look out for the opportunity to commit acts of beneficence, lest you become someone who says they care but whose actions indicate otherwise (cf. Buchanan, 1996, p. 31).

My normative intention with the all-stakeholders-considered case for corporate beneficence is to verbalize a framework of practical reason that management might find useful in ascertaining its own first-person sense of responsibility, given the personal commitment to beneficence that, by stipulation of the account I am developing, they embrace (Table 1). What management can *afford to* do, all stakeholders considered, has to be ascertained by management’s subjective sense-making. This concept becomes definite and prescriptive when management concludes that the act of beneficence being mooted falls squarely on the side of what the corporation *can* afford to do, consistent with management’s role responsibility helming the ship of the corporation, *all stakeholders considered*.

### The mRNA Case for Corporate Beneficence

The present pandemic has left millions dead. Billions remain unvaccinated—in wealthier nations this may be due to disinformation and misguided politics, but poorer nations lack access to the mRNA vaccines that have proven so effective in preventing serious illness and death from COVID. Many questions about corporate beneficence could be stated—I started the article asking about the companies Moderna and Pfizer–BioNtech that control the miraculous vaccines. According to my normative approach, any insights into the moral forces that may exist to compel these three companies to reconsider the hard line they are currently taking depends on what their management actually cares about. In the absence of such data, I will demonstrate how my account works by looking at these companies’ mission statements as proxies for what management cares about.

Dunfee (2006) himself observes that the mission statements of pharmaceutical companies “express in general terms a commitment to enhancing human welfare. This is particularly true of the firms in the global pharmaceutical industry which have, by and large, recognized a fundamental commitment to improving human well-being” (p. 190). For example, Merck, renowned in business ethics for the river blindness case, holds that: “We aspire to improve the health and wellness of people and animals worldwide, and to expand access to our medicines and vaccines” (Merck, 2019). What is striking about the three companies in question is that only the mission statement of Pfizer, alone a longstanding global leader, suggests beneficence: “We innovate every day to make the world a healthier place” (Pfizer, 2021). Moderna and BioNtech have missions that foreground technology over health. For Moderna, the mission is “Deliver on the promise of mRNA science to create a new generation of transformative medicines for patients” (Moderna, 2021). For BioNtech “Our mission is to develop the next generation of immunotherapies to improve clinical outcomes for patients and usher in a new era of individualized medicine (BioNtech, 2021).

If Moderna as a company was founded for technology development, first and foremost, and its management do not bring a strong enough personal commitment to beneficence to override that intention, then it is perhaps to be expected that management would not choose to prioritize saving lives now over developing new drugs in the future. This orientation could explain why Moderna (“create a new generation of immunotherapies”) has not made its vaccine available to low income countries but Pfizer (“make the world a healthier place”) has (Robbins, 2021). In any case, the apparent difference between the missions of the companies means that the public has an argumentative fulcrum to press Pfizer to do more that does not exist with Moderna. Stakeholders can remonstrate to Pfizer management to explain how they can continue to say their mission is to make the world a healthier place when they don’t do more to expand access to their mRNA vaccine.

Recall what happened when the governments of South Africa and India, with the support of NGO groups, pleaded for pharmaceutical companies to provide royalty-free licenses to manufacture patented antiretroviral drugs 20 years ago (Chance & Deshpandé, 2009). The cost of granting a royalty-free license of patents is hardly material in the scheme and scope of a global pharmaceutical firm’s activities, roughly limited to the time internal and external legal counsel and their managerial counterparts spend on the licensing agreement (since there is no manufacture or distribution required). When the problem faced by the requesting countries is that their infected population fails to register economic demand to survive at the going $10,000 per capita per year price for triple-cocktail therapy, the company would not be squandering revenue by granting the royalty-free license.^19^ The plea for intellectual property during the HIV pandemic fell upon the ears of managers who ultimately concluded they could afford to grant the licenses (Pogge, 2020), and if personally committed to beneficence (as suggested by company mission statements), I have argued they would have chosen inaction—refusing a reasonably circumscribed royalty-free license—at the peril of corrupting their personal integrity and sense of beneficence. Supposing they in fact cared about promoting the well-being of humanity, this opening to legitimately save numerous lives with the mere grant of a limited license would be unusually compelling. In fact, the management of several major pharmaceutical companies acted consistently with the foregoing analysis to license their HIV drug patents royalty-free, including to Aspen Pharmacare, a low-cost manufacturer that produces the medication at prices the Clinton Foundation was willing to pay in South Africa, the country with the most HIV-infected persons (Chance & Deshpandé, 2009).

The key feature of the all-stakeholders-considered case for corporate beneficence is that it depends on the commitments of the moral agents in question. The case of the mRNA vaccines suggests that, at least with respect to tech-focused companies like Moderna and BioNtech whose management may *not* embrace a personal commitment to beneficence—or they might consider that, in light of the stated corporate mission, their responsibility *qua* management limits their discretion to sway from the priority of new drug development—public health depends upon public policy because beneficence may not of its own force bring management to act.^20^ Fortunately, for many managers at public and private companies, a commitment to beneficence is bound up in their conception of selling products and services to the public (e.g., Simpson, 2020). The COVID pandemic revealed how quickly managers who cared and saw the opportunity to do so redeployed corporate capabilities in service of pressing human needs, from car companies producing ventilators to beverage companies making hand sanitizer (Albergotti & Siddiqui, 2020; Tiernan, 2020).

## Limitations

The form of my inquiry in this article and, therefore, its arguments and conclusions are subject to several important limitations. Though I review several moral forces in the literature linked to the deployment of corporate capabilities towards acts of beneficence, I do not claim that the taxonomy outlined (see Table 1) is exhaustive. For example, Jones and Felps (2013) develop a “neo-utilitarian” objective they claim should motivate management’s engagement with stakeholders. It is beyond the scope of this article to assess the extent to which Jones and Felps’s position (or variations thereon) resemble the all-stakeholders-considered case for corporate beneficence set forth herein.

With respect to the development of the all-stakeholders-considered case, it is important to note certain simplifying assumptions used for the analysis. First, the collective who form “management” is assumed to be made up of people, but as people the account takes them to be monolithic in their embrace of the duty of beneficence. In reality, managers have different viewpoints and engage in internal dialog and process that is pivotal in leading corporations to deploy capabilities (or not) towards acts of beneficence (see Bower, 1970; Bower & Christensen, 1995); however, I abstract from those differences to theorize about the simpler, idealized case. Moreover, I do not attempt to relate my analysis to debates about collective responsibility and corporate moral agency (MacArthur, 2019; Orts & Smith, 2017; Scharding, 2019). Further research should be pursued to bring light to both sets of limitations—comprehensiveness of the taxonomy and simplifying assumptions of the analysis. Moreover, empirical research should examine the proposition that managers who are committed to beneficence and face propitious circumstances to save lives, all stakeholders considered, experience an internal force that drives them to want to deploy corporate capabilities accordingly.

## Conclusion

My account of the moral imperative to commit acts of corporate beneficence that is yielded by the all-stakeholders-considered case extends the beneficence literature in two important ways. The moral imperative is motivated and driven by the imperfect duty to promote the well-being of others, as in prior literature, but by pushing the Kantian logic of duty in the managerial context from the first-person point of view, I gain the traction needed to establish a principle that compels corporate action for stakeholder-oriented managers who are personally committed to beneficence. The force of the imperative given by this account comes from within management’s personal commitment to beneficence and integrity: that joint commitment exerts force against the incongruence of inaction. Crucially, this moral imperative becomes operative in the class of cases that support management’s judgment that the corporation can afford to act,^21^*all stakeholders considered*.

The form of my account differs markedly from scholarship in business ethics whose normative intention is typically directed towards companies and managers *in the abstract*, which is to say that the beneficence literature has not hinged the normative inquiry on the factual question of whether managers are actually personally committed to promoting the well-being of others. Whereas managers’ personal values are mostly left in the background by normative business ethicists, Friedman (1970) expressly recognizes that:

the corporate executive is also a person in his own right. As a person, he may have many other responsibilities that he recognizes or assumes voluntarily—to his family, his conscience, his feelings of charity, his church, his clubs, his city, his country. He may feel impelled by these responsibilities to devote part of his income to causes he regards as worthy, to refuse to work for particular corporations, even to leave his job, for example, to join his country's armed forces. If we wish, we may refer to some of these responsibilities as ‘social responsibilities’ (p. 33).

However, Friedman highlights the existence of executives’ personal values so he can be crystal clear that management ought to leave those personal values at home; for example, he claims it is a moral trespass to “make expenditures on reducing pollution beyond the amount that is in the best interests of the corporation or that is required by law in order to contribute to the social objective of improving the environment” (p. 33).

My inquiry into the moral forces that compel corporate beneficence is not impartial to what management cares about, but rather zooms in to theorize about management that *does* embrace a personal commitment to promote the well-being of others. This boundary condition might appear to limit the value of my account. Certainly, if one’s objective is to define concrete standards that should apply to all corporations, regardless who manages them, my approach will not do. In concluding, I would like to explain why I see the empirical contingency embedded in my account as a strength, rather than a weakness.

First, my theorizing avoids moralizing—telling management what they should care about (even if they don’t). Rather, the structure of the account creates space to “preach to the choir” of managers who embrace a commitment to promote the well-being of others, like Hubert Joly, Best Buy’s former chairman and CEO:

My individual, personal purpose is to try to make a positive difference for people around me and then to use the platform I have to make a positive difference in the world. This is an evergreen purpose, meaning, whether I’m the CEO of Best Buy or starting my next chapter, it’s always true (Simpson, 2020).

Sharing a similar sentiment, the CEO of the Harpoon Brewery said that in the early days of the pandemic he was “trying to pay attention and find ways we can help,” and that is why the company switched from making beer to hand sanitizer in 2020 (Tiernan, 2020). Thus, the empirical contingency built into this way of theorizing invites managers to explore their values rather than judging them.

My core claim is that management’s moral commitment, its sense of beneficence, has the capacity, within a managerialist, stakeholder-oriented conception of corporate governance, to serve as a prime mover in its own right to deploy corporate capabilities beyond the business case in responding to urgent human needs. In the picture that emerges, the potential costs and benefits of acts of beneficence that dominate the business case manifest as weights on the scale that more or less supports or frustrates management’s judgment that it can afford to act on behalf of the corporation. Under this alternative moral logic, business case arguments serve to loosen constraints on the capacity of the company to do good beyond the market, and also through markets, given that sales constituting acts of corporate beneficence provide a rich outlet for managers to realize their sense of moral duty, all stakeholders considered.

In considering the mRNA vaccine owners, I suggested that if management in fact aligns with the companies’ respective mission statements, then Moderna and BioNtech management may not have a personal commitment to promoting the well-being of humanity strong enough to override what is apparently most advantageous for technology development, i.e., *not* sharing the formulas right now. Maybe Pfizer is different, or maybe in its partnership with BioNtech, Pfizer is also more geared to innovation than public health (Kaplan et al., 2010). To the worry that my approach is bound to yield slippery and uncertain guidance to the public, my reply is that the primary target—besides scholars—is the people making decisions on behalf of these companies. Have they asked themselves what their companies can afford to do? Or have they been locked into a business case mentality (Kaplan, 2020; Taylor, 2017)? Have they asked themselves whether they embrace a commitment to promoting the well-being of humanity and what latitude their sense of role responsibility may allow to actually do so? My hope is that this article’s analysis will catalyze managers to rethink their personal priorities and also their conception of corporate governance in a manner that clears the way for corporate beneficence. Even if it does not, I have sought to provide scholars with greater clarity about the moral forces that may or may not be forthcoming (and why or why not) to unleash corporate capabilities in service of the well-being of humanity.

## Footnotes

1 As a matter of fact, government funding was a precondition to the success of the two mRNA vaccines. In footnote 20, I touch upon the possibility that the $6 billion spent by United States taxpayers (Saltzman, 2021) provides grounds to press Moderna and Pfizer–BioNtech to relax their legal entitlement to fully capitalize on the patent rights that the World Trade Organization’s Treaty on Trade-Related Aspects of Intellectual Property (TRIPS) transforms from a national right—as with nearly every other category of patent, including biotechnology manufacturing—into a global blockade on making lifesaving pills. At present, Moderna has taken what strikes the author as a cynical posture—announcing they will decline to exercise the right to block the manufacture of the mRNA vaccine at the same time they hide a script known to prevent death (Roelf, 2021).

2 According to Harrison et al. (2019), proto-Chicago Berle conceded by the 1950s that society had declared Dodd the debate’s winner for “supporting a broader purpose that includes secure employment, quality products for customers, and contributions to the good of society” (p. 4).

3 Recently though a surge of companies like Uber promote shareholder value profitlessly by capturing market share first (Horan, 2017).

4 In the analysis that follows, I focus on whether and why acts of corporate beneficence may become, in Mejia’s (2021b) terminology, non-discretionary. His analysis of cases where agent’s duty has no latitude but does enjoy discretion (pp. 436–444) is responsive to the needs of his account which evaluates how shareholders’ duties do or do not carry through to management. These complications are not required by a managerialist account and, moreover, my inquiry in this article is into the moral motivation to commit *acts* of beneficence. Finally, I follow Hill’s (1971) authoritative account of imperfect duties which does not utilize Mejia’s distinction between latitude and discretion.

5 For this reason Mejia (2021b) recognizes that moral duty “allows the agent to appeal to her inclinations, passions, and sensibilities to determine whether to fulfill the duty on a particular occasion” (p. 428).

6 I appeal to Hill’s formulation rather than the specific conditions Mejia defines for the duty of rescue because it is more generic. The duty of rescue as defined by Mejia (and Dunfee) represents a class of cases that fit within Hill’s general formulation.

7 An original conceptualization of beneficence by Mansell (2013) imagines (contra Friedman’s (1970) stipulations about their desires) that shareholders might embrace the duty of beneficence and, therefore, wish managers to act accordingly (but see Mejia, 2021a, 2021b). By according primacy to shareholders but enhancing the reach of their desires, Mansell supports as compatible with his sense of shareholder primacy a conception of stakeholder theory “which allowed a particular corporation in specific circumstances to pursue as an end the interests of some nonshareholders, and thus permitted the objective to vary according to shareholder discretion” (p. 597). The interesting question of how Mansell’s view might dovetail with and extend Dunfee’s (2006) account of the SMMO falls outside the scope of this article.

8 Bowie’s framing of beneficence is suggestive of Porter and Kramer’s (2011) call on management to “create shared value” by formulating long-term oriented corporate strategy to achieve competitive advantage that is responsive to social and environmental needs.

9 The combination of three kinds of ARVS was discovered in 1996 to delay the onset of AIDS for more 5 or more years. Today triple therapy can delay AIDS for more than 20 years (Vella et al., 2012).

10 Remarkably, Dunfee (2006) avoids all reference to the word “beneficence” in his article. I would speculate that he found the duty of beneficence inadequate for his goals, given its treatment by business ethicists. In any case, as Kant’s morality and political theory position his thought in the social contract tradition, Dunfee’s approach may be seen as relatively continuous conceptually with the beneficence literature. I thank an anonymous reviewer for this observation.

11 In section “The All-Stakeholders-Considered Case for Corporate Beneficence” of his article, Dunfee speaks to the question, and yet the closest he gets to giving a moral intuition for why corporations should, as a matter of an extant social contract, have to do something substantial rather than nothing at all in such cases comes only from an analogy to a hypothetical doctor who witnesses children who are allergic to bee stings being stung. The difficulty with this strategy is that it does not address the difference between a corporation’s social contract and individual people, when the force of the imperative must be heard by managers, whose professional responsibilities are shaped, at least in part, by the corporate form (see Bower & Paine, 2017). A manager, operating through a large corporation, does not have the same leeway to act and calling that faces a doctor on the street.

12 In the case of a corporation that already has a social investments budget, Dunfee’s argument enjoys the intuition that I develop in this section (i.e., management can afford to do good). Under conditions (3) and (4) in the second sentence of the SMMO, the moral appeal of the obligation shifts from “do what you can afford to do” to: “do what you *should be able to* afford to do.” However, Dunfee does not explain why this norm should exist, beyond saying that “in fact many firms express in general terms a commitment to enhancing human welfare” (p. 190). But not all companies do, and one of the premises of ISCT is the right of economic actors to exit from “microsocial communities” (Donaldson and Dunfee, 2002, p. 1857).

13 Thanks to the anonymous reviewer who suggested the pertinence of Jones (1991) to the limitations of Dunfee’s account and as an area for further research into the strength of the all-stakeholders-considered case for corporate beneficence.

14 Dubbink (2018) argues persuasively that Dunfee’s effort to turn the rescue duty into a kind of “voluntary tax” undermines the plausibility of his framing of the SMMO (the SMMO stops short of requiring any actual rescue and if the contribution in question operates like a tax, why should it be limited to companies with unique competencies?): The idea of such a voluntary tax [the SMMO] seems to have been made with the overdemandingness problem in mind: a small percentage of profits can never be overdemanding. The problem with this solution is that the idea of an obligatory annual gift is out of touch with the idea of being a bystander on account of the duty of rescue. An agent who has to acknowledge the duty of rescue asks themselves: what is there to do here and now? It is also out of touch with the personal perspective on morality presupposed by the idea of a bystander. Given the personal perspective, it is important to demonstrate the relation with direct, required action that has to be performed, here and now. The tax has nothing of that kind. It is more like an anonymous and pseudo institutional solution broken free from the personal perspective. One of the ways in which this shows is that it is hard to argue why only ‘uniquely positioned’ bystanders must pay this money. If it all comes down to paying money, there is no rationale for the requirement that the bystander must be uniquely positioned (p. 9). Central to the all-stakeholders-considered case for corporate beneficence that I develop in section “The All-Stakeholders-Considered Case for Corporate Beneficence” is moving away from the sense of an abstract compliance obligation imputed by the SMMO to management’s internal point of view and capacity to make a difference by virtue of the corporation’s comparative advantage to respond to the catastrophe at hand.

15 For example, in specifying a minimal obligation pegged to the social investment budget, Dunfee’s premise is that, to comply with its minimal obligation, management may divert resources from current beneficiaries of corporate social investments. The SMMO does not require a company to ratchet up its total contribution to social causes any time the obligation ripens with a human catastrophe calling for the company’s capabilities. This prompts the third sentence, which is responsive to shareholder interests and also anticipates the concerns of the current beneficiaries of a company’s social investment activities (Table 2, Section VI). What if, despite the salience of a human catastrophe, existing beneficiaries make an equally compelling call for the corporation’s capabilities (Mitchell et al., 1997)? Avoiding imposing on shareholders, Dunfee strikes a compromise between the pair of non-market callings, slackening the SMMO to allow the resources earmarked by the SMMO to be split between old and new stakeholder contributions.

16 The pharmaceutical industry’s broad commitment to corporate responsibility initiatives to respond to the HIV crisis beyond the paying market by the time Dunfee was writing (as he documents in Section “Dunfee’s Statement of Minimal Moral Obligation”) constituted a dramatic reversal of its earlier political strategy, which famously featured a high-profile, reputation-tarnishing lawsuit against South African President Nelson Mandela that was eventually dropped (Chance & Deshpandé, 2009).

17 To be sure, win–win approaches like Creating Shared Value (Porter & Kramer, 2011) might respond to human needs and generate profit. Dunfee’s assumption (not material to my argument) is that the social investment budget *qua* social investment budget is not deployed for profit-making initiatives.

18 In what spaces and in what ways do or should management engage stakeholders to assess questions like what the corporation can afford, all stakeholders considered? These open-ended, sense making inquiries can access the diversity of ways that the corporate governance of companies may engage with stakeholders, including the following categories recently highlighted in the *Academy of Management Review* (Amis et al., 2020): • Stakeholder governance as a bargaining game among stakeholders. • Stakeholder governance as creating forums where conflicts among stakeholders can be addressed. • Stakeholder governance through establishing priorities among different stakeholders. • Stakeholder governance as a process of finding ways to resolve stakeholder conflicts. What is, perhaps, possible to say without qualification is that: Solving tough issues together builds trust and fosters partnership. And isn’t it wiser to have critics in the room, working productively, than outside the building, holding demonstrations and hanging banners in protest?” (Polman & Winston, 2020, pp. 129–130).

19 The exception would be due to the risk of gray market imports that could cannibalize a company’s full-price market in the United States and other wealthy industrial countries.

20 United States taxpayers invested $6 billion in Moderna (Saltzman, 2021)—advocates for global public health may find better traction arguing to the federal government that it should either pressure these companies directly or instead focus on convincing fellow members of the World Trade Organization to waive patent rights on the mRNA vaccines pursuant to the Treaty on Trade-Related Aspects of Intellectual Property (Maxmen, 2021; Robbins, 2021; Roelf, 2021).

21 This formulation appears to be in tension with Ohreen and Petry (2012) who hold that “a Kantian moral duty of beneficence should not factor in non-moral considerations (such as avoiding taxes or increasing profitability through public exposure) in determining the required degree of beneficence” (p. 375). I have two tentative replies. The first is that there is no tension since my account puts the moral judgment in *can’t afford not to* (which evaluates the congruence of moral commitments in the will) rather than *can afford to* (which arguably evaluates “non-moral considerations”). The second reply would be that avoiding taxes and increasing profitability are moral considerations, so the principle at issue is unclear.
